# Perspective on salutogenic approaches to persistent pain with a focus on mindfulness interventions

**DOI:** 10.3389/fpain.2023.1188758

**Published:** 2023-08-29

**Authors:** Carole A. Paley, Mark I. Johnson

**Affiliations:** ^1^ Centre for Pain Research, Leeds Beckett University, Leeds, United Kingdom; ^2^ Academic Unit of Palliative Care, University of Leeds, Leeds, United Kingdom

**Keywords:** mindfulness and chronic pain, salutogenesis and persistent pain, painogenicity, mindful approaches to pain, whole health, ecology of wholeness

## Abstract

In this article, we provide a unique perspective on the use of mindfulness interventions in a whole health framework embedded within the theory of salutogenesis and the concept of painogenic environments. We argue that mindfulness is a valuable tool to bridge exploration of inner experiences of bodily pain with socio-ecological influences on thoughts and emotions. We outline research from neuroimaging studies that mindfulness techniques mediate neural processing and neuroplastic changes that alleviate pain and related symptoms. We also review evidence examining behavioural changes associated with mindfulness meditation providing evidence that it promotes self-regulatory activity, including the regulation and control of emotion and catalysation of health behaviour changes; both of which are important in chronic illness. Our viewpoint is that mindfulness could be a core element of salutogenic approaches to promote health and well-being for people living with pain because it rebuilds a fractured sense of cohesion. Mindfulness empowers people in pain to embrace their existence; shifting the focus away from pain and giving their lives meaning. We propose that integrating mindfulness into activities of daily living and individual or community-based activities will promote living well in the modern world, with or without pain; thus, promoting individual potential for fulfilment. Future research should consider the effects of mindfulness on people with pain in real-life settings, considering social, environmental, and economic factors using a broader set of outcomes, including self-efficacy, sense of coherence and quality of life.

## Introduction

Painogenicity, described as the tendency of socio-ecological environments to promote persistent pain (1), and salutogenesis, a concept that considers the origins of health as opposed to the origins of disease, have proved useful ways of exploring a healthy settings approach to the challenge of persistent pain in society (2–5). Salutogenesis is premised on the concept of a sense of coherence—the way people make sense of the interaction of their body in the world—and is fundamental to understand why some people develop persistent pain whilst others do not (6, 7). Sense of coherence operates at individual, group (family), organization and societal levels and includes *meaningfulness* of one's life, *comprehensibility* of stimuli arising from the internal and external environments, and *manageability* of these stimuli using resources at a person's disposal, such as health care services and treatments, social networks and peer support, and self-coping strategies to promote mental and physical well-being (8).

Mindfulness involves paying non-judgemental attention to experiences inside and outside of “oneself” on a moment-by-moment basis to aid reconnection of sensations, thoughts and feelings with the outside world in a positive way (9). Our viewpoint proposed here is that mindfulness is a valuable tool to bridge exploration of inner experiences of bodily pain with socio-ecological influences on thoughts and emotions. In this article, we discuss mindfulness interventions within the framework of the theory of salutogenesis and the concept of painogenic environments.

## Shortcomings of the biopsychosocial model

The biomedical model, which associates pain with potential or actual tissue damage, has, at least in part, fostered a reductionist and materialist approach to alleviating pain by analysing and diagnosing the status of tissue, rather than synthesising factors affecting a whole-person's lived experience of pain in the complex socio-ecological milieu of the modern era (10–12). In recent decades, the shift towards a broader biopsychosocial model of pain has acknowledged the importance of psychosocial risk factors—employment conditions and socioeconomic status as two examples—which potentially promote pain and hinder recovery resulting in pain management strategies utilising multimodal interventions and multidisciplinary teams. Often, biopsychosocial interventions are delivered as discrete entities targeting specific elements of a person's psychophysiology in a disconnected manner (e.g., surgery, medication, exercise, diet therapy, cognitive behavioural therapy (CBT), acceptance and commitment therapy (ACT), etc.) Thus, some people experience fragmented care to the detriment of their health and well-being. Moreover, there is a treatment-prevalence paradox in which an ever-increasing variety of interventions have not reduced the burden of persistent pain. Calls to shift emphasis towards holistic models of pain supported by integrated health care service delivery are growing (13, 14).

## The whole health model

Gaudet advocates a cultural transformation of *the purpose* of healthcare, and other systems impacting on health and well-being, underpinned by the concept of “whole person health” (15). Gaudet argues for a change in the focus of health care by discovering what gives people a sense of meaning and purpose in their lives and building systems that support this. Advocates of Whole Health view a person's life as a journey of “push and pull” within a continuum of health and disease. Thus, Whole Health focuses on factors that create health not just factors that prevent disease.

The concept of wholeness has been discussed by academics for decades; mostly emanating from the “Whole Person Medicine” movement during the 1970's which had its roots in complementary and alternative medicine (CAM) (16). Viewpoints from different CAM protagonists have recently been discussed (17), providing a summary of current thinking from academics endorsing a variety of approaches. Interestingly, it is acknowledged that this model is still falling short of providing effective, integrated care, even after decades of research because “… *in order to successfully transform the existing biomedical model, the Whole Health model must demonstrate validated research outcomes* …” (17) (p. 3). This, according to Langevin, needs to be achieved by moving away from the reductionist approach to biomedical research (18) and there is still much debate on how this can be achieved.

In 2020, Agarwal published a postmodernist, social constructionist treatise on an “ecology of wholeness” which describes the relationship between the biomedical understanding of the body in pain and its relationship with a person's awareness of themselves and their interaction with micro and macro level aspects of material existence with the natural world (19). Agarwal's ecological model of wholeness comprises The Self (i.e., reflexive and embodied) and The Body [i.e., material and discursive (conversational)], in relationship with The Context (i.e., time, change, Illness intrusion, traditional health system, food, nature, body/self-integration) (19). Agarwal's model emphasises the need to consider not only the person within a diagnostic framework, but also the wider ecological (salutogenic) setting (19, 20). Within this framework, patient involvement, education and empowerment are central tenets.

We advocate reconfiguring the health care mindset away from a reductionist and materialist viewpoint towards a whole-person (societal) health lens that focuses on factors that support and empower people to create their own health and wellbeing, i.e., salutogenesis (2, 5).

## Pain and salutogenesis

**Figure 1:**
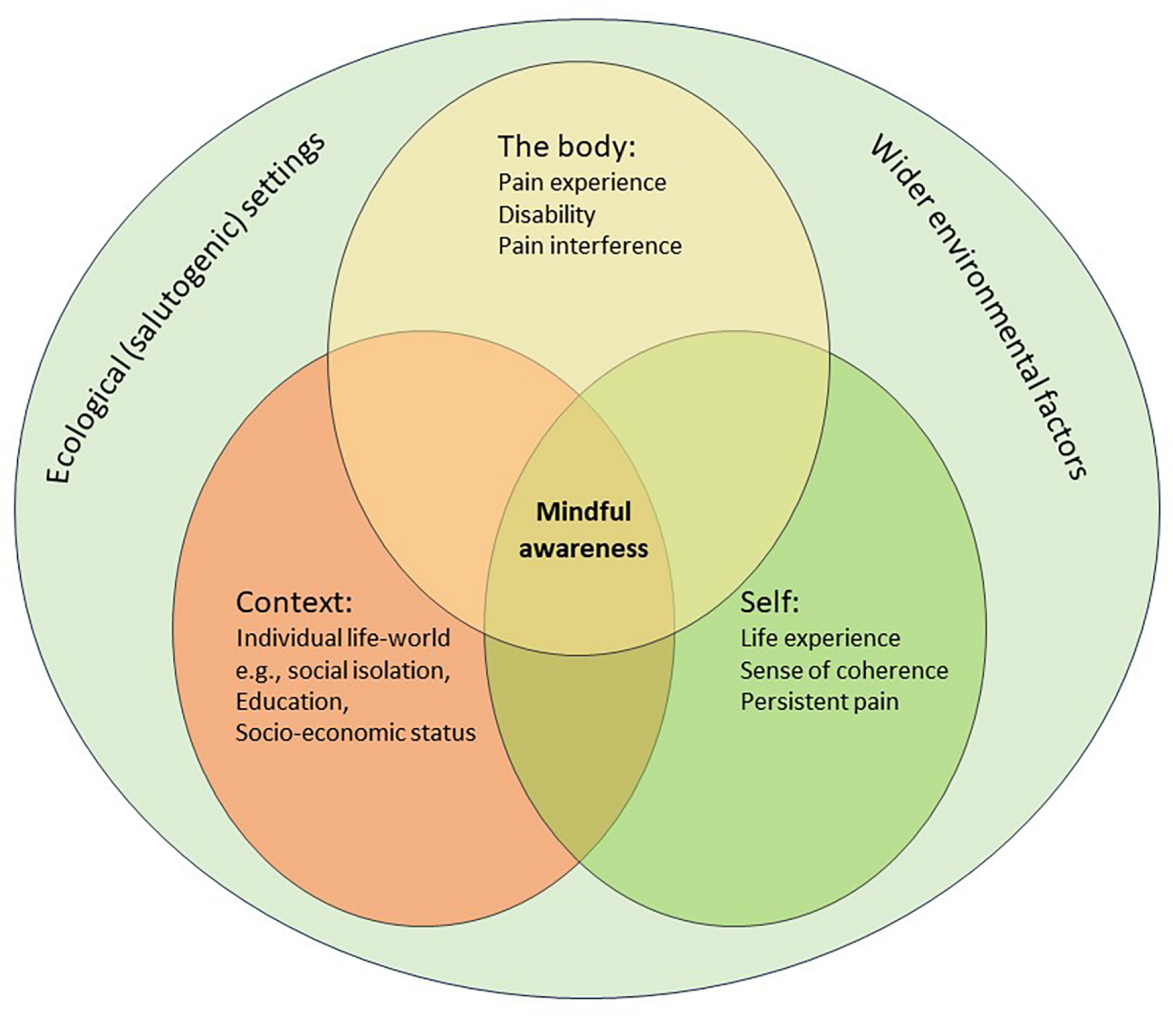
A whole-health model of mindful awareness within a salutogenic framework for individuals with persistent pain (19).

In 1979, Antonovsky introduced the term “salutogenesis”, meaning, “the origins of health” (21). It considers the origins of health as opposed to the origins of disease (22). The key to understanding persistent pain within a salutogenic framework is to consider how people can develop meaning from their suffering and translate pain into something positive and meaningful for them through their internal sense of coherence, either as an individual or as part of a group (7). Salutogenesis is influenced by surroundings, socio-economic and environmental factors that can promote or hinder recovery from pain. Its premise is that people can be healthy despite pain. Environments which promote the persistence, severity, or impact of pain, including hindering recovery from pain, have been described as “painogenic” (1). The relationships between the salutogenic framework and Agarwal's “ecology of wholeness” in a context of persistent pain and whole health are illustrated in Figure 1.

## Painogenicity

The term “painogencity” was introduced to reflect parallels between persistent pain and Boyd Swinburn's concept of obesogenicity (1). Swinburn defined obesogenicity as the sum of influences that the surroundings, opportunities or conditions of life have on promoting obesity in people or populations (23). It moved away from an individualistic understanding that obesity was “caused” through individual choice or action towards a wider recognition that social, environmental, and commercial conditions may promote conditions that made obesity more likely. Johnson (1) appraised persistent pain from an evolutionary mismatch-perspective and argued that, like obesity, socio-ecological conditions may promote the persistence of pain i.e., that aspects of modern-day living were painogenic. Thus, painogencity was defined as the tendency of socio-ecological environments (or “settings”) to promote persistent pain.

## Settings-based approaches and health promotion

Rather than focusing on the manifestation of a behaviour or condition (i.e., pain), academic debate on alternative ways to approach the problem of persistent pain, using interventions embedded within the theory of salutogenesis and a settings approach to health and wellbeing has become more accepted in the discourse (7, 24, 25). It is grounded in the World Health Organisation's (WHO) Ottawa Charter, which prioritised empowering people and communities to increase control over, and to improve, their own health by providing the conditions and resources to do this (26).

Settings-based approaches to health are “upstream” and maximize disease prevention and its impact on health (e.g., pain) by attending to the settings where people actively use and shape their environment and create or solve problems relating to their health. Settings comprise physical boundaries and organisational structures. They include homes, workplaces, schools, villages, towns, cities, hospitals, prisons etc. The goal of a settings-based approach is to maximise health promotion and disease prevention through a “whole system” model of public health via community participation, partnership, empowerment and equity (26, 27). Empowering people to understand and make sense of their lives by increasing their sense of coherence has been shown to decrease the risk of non-communicable diseases for which there are currently programmes acting solely on “downstream” risk factors (e.g., hypercholesterolaemia, hypertension and type 2 diabetes) (28).

Campaigns to enable people to explore their emotional relationship with pain and to offer strategies to foster living better lives with their pain have arisen; e.g., *Live Well with Pain* (https://livewellwithpain.co.uk/), *Pain Café* (https://pain.cafe/), *Flippin' Pain* (https://www.flippinpain.co.uk/), *Pain revolution* (https://www.painrevolution.org/), and others*.* These strategies include, amongst other things, mind-body techniques that increase a person's sense of coherence, such as mindfulness meditation, yoga, tai chi, relaxation techniques and hypnosis. Of these techniques, mindfulness has risen in popularity since the end of the 20th century with healthcare providers (29). Integrating mindfulness practice into activities of daily living can improve a person's sense of coherence.

## Mindfulness

### Context

The historical roots of mindfulness date back to the 5th century BC. Mindfulness has been defined as: “*…the awareness that emerges through paying attention on purpose, in the present moment, and nonjudgmentally to the unfolding of experience moment by moment*” (30). Mindfulness involves people being aware of their situation, without being reactive, judgemental, or overwhelmed by what is happening to them or around them. Mindfulness interventions in various guises have been used to prevent or alleviate maladaptive perceptions of pain, such as catastrophising and associated psychological manifestations such as anxiety and depression (31).

The principles of mindfulness can be readily incorporated into creative activities (e.g., music, drawing, writing, painting, clay making, dance), daily activities (e.g., shopping, washing dishes, gardening, walking) and sport, recreation and exercise activities (e.g., swimming, fishing and yoga), both individually and as a group. The optimal state of mindfulness is of “relaxed alertness”, which is associated with better mental health (32). Interestingly, it is likely that people who are able to withstand extreme physical discomfort, such as ultra-endurance athletes (33) or those engaging in extreme sports (34) are able to enter some sort of mindful, or self-hypnotic state to divert their attention away from the physical (and psychological) pain.

### Beneficial effects of mindfulness

Comprehensive reviews of evidence suggest that mindfulness alters processing of multiple brain regions leading to a variety of beneficial effects for people with persistent pain (35–37). Briefly, mindfulness practices involving focussed attention (e.g., slow, rhythmic breathing or body scanning techniques), promote calmness and relaxation that increase parasympathetic activity (vagal tone) which ameliorates the hypothalamic-pituitary-adrenal (HPA) axis response to stressors such as pain. This improves physiological status including blood pressure, respiration, heart rate reactivity, fatigue and pain and other bodily sensations (38–41). Mindfulness decouples thalamus–precuneus and ventromedial prefrontal deactivation, effectively inhibiting onward transmission of nociceptive input (42). Mindfulness improves emotional and cognitive well-being in people with persistent pain mediated in part by functional alterations in the insula, amygdala, and hippocampus (35–38, 42–50). Neuroplastic changes occur in the insula associated with interoception and a reduction in negative emotional responses to unpleasant sensations such as pain (40, 51). For some people, patterns of neural activity experienced during mindfulness practise can be replicated at will (52). Thus, better acceptance of painful or unpleasant sensations are achieved through modulation of negative appraisals of interoceptive stimuli and by promoting coping strategies (40, 53, 54).

A review of evidence examining behaviour changes associated with mindfulness meditation provided evidence that the intervention promotes self-regulatory activity, including the regulation and control of emotion and catalysation of health behaviour changes; both of which are important in chronic illness (55). Other behavioural changes such as self-compassion are also thought to occur; encouraging behaviours associated with self-compassion and a reduction in overidentification with painful experiences (56). A meta-analysis also showed beneficial effects on negative self-related rumination, suggesting that it might reduce repetitive focus on symptoms (57).

Clinical research evidence for beneficial effects of mindfulness interventions for people with persistent pain is growing, but based on small, under-powered studies which show statistical significance but fail to demonstrate favourable effect sizes (35, 58, 59).

A systematic review published in 2016 indicated that mindfulness produced small improvements in pain symptoms based on a meta-analysis of 24 RCTs of low quality (60). systematic review of 30 RCTs published in 2017 found that mindfulness produced small improvements in the severity of persistent (chronic) pain compared with various control groups (61), yet a systematic review of 13 RCTs published in the same year found that mindfulness did not improve the severity of persistent pain but did improve psychological aspects of pain, such as depression (62). The methodological quality of RCTs included in both reviews was judged to be low. In 2021, Pei et al., conducted a systematic review of eight RCTs that did not find any statistically significant differences between mindfulness and control groups on the severity of persistent pain, although there were improvements in mindfulness and depression in the short-term (63). Pei et al., suggested a need for evaluations of dose–response to optimise mindfulness technique. We suggest that these systematic review findings are promising and might provide impetus for further research.

Since the COVID-19 pandemic, online and smartphone self-help interventions for pain, including mindfulness techniques have become popular (64), but research findings on the efficacy and effectiveness of these self-help interventions are inconclusive (65). However, these applications potentially represent a cost-effective way of implementing mindfulness interventions.

Reviews and evidence syntheses of mindfulness studies for persistent pain conditions using qualitative methodologies were not found but several small individual studies of varying methodological quality reveal several recurring themes following mindfulness-based interventions. A small analysis of four groups of older adults with persistent low back pain, following an eight-week mindfulness programme revealed benefits such as overcoming fear of pain, a reduction in negative emotions and a reduction in focus on the pain (66). Another demonstrated an improvement in pain-related strategies following group mindfulness and problem-solving (67), and a feasibility study found patients more empowered to look after themselves, and were more self-aware and in the moment following an eight-week Mindfulness-Based Stress Reduction (MBSR) programme (68).

**Figure 2:**
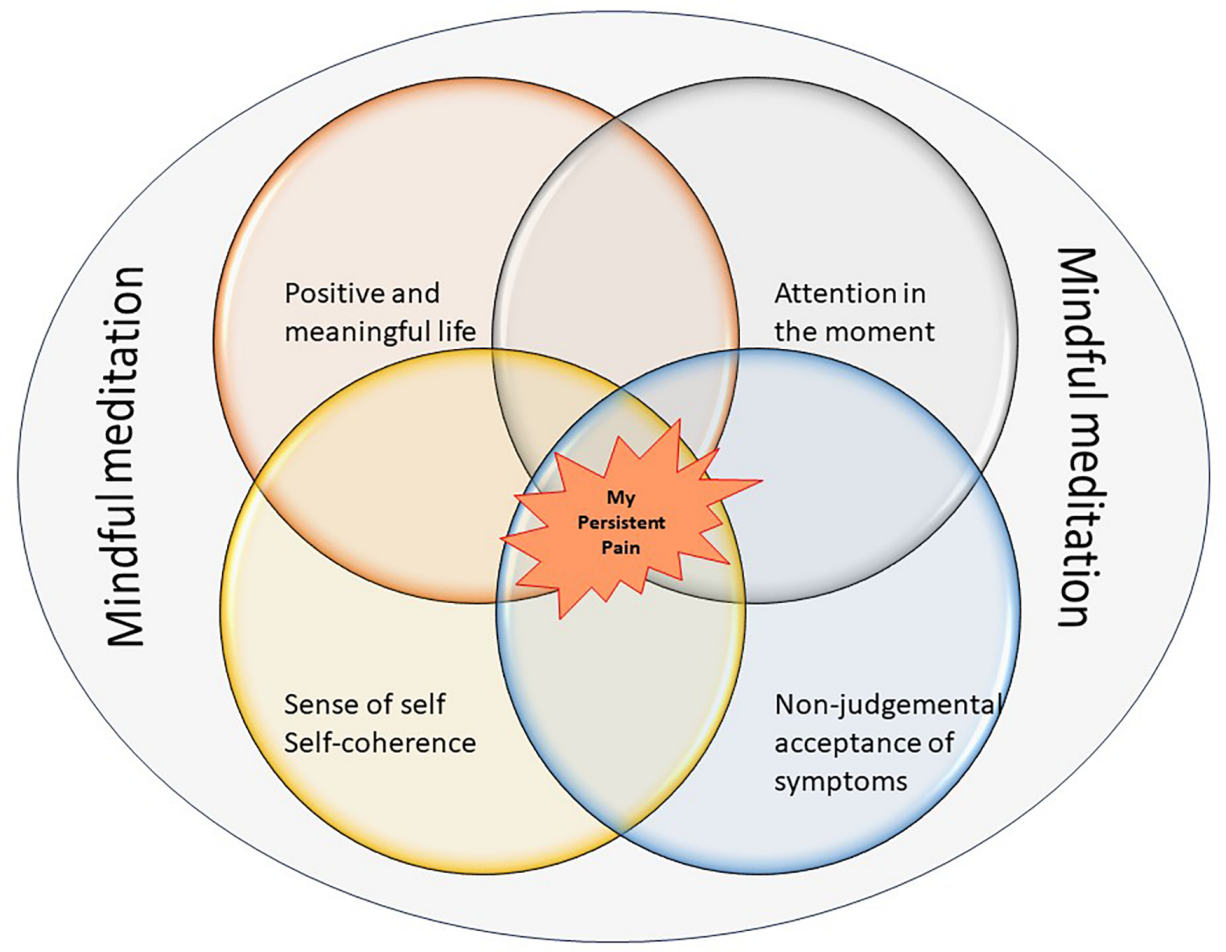
Outcomes of mindfulness interventions for persistent pain.

A simplified diagrammatic illustration of the overlap between the key elements of mindfulness meditation in the context of persistent pain is shown in Figure 2.

### Adverse effects of mindfulness meditation

As with any intervention, an awareness of possible adverse effects is essential but frequently overlooked. The British Psychological Society guidelines on mindfulness-based approaches warns that these interventions should not be expected to confer benefit on everyone (69), although the prevalence of adverse events is thought to be no more likely than it is using other psychotherapeutic techniques (70).

Inducing body-awareness and introspection through mindfulness can vary within and between people and may produce detrimental effects such as autonomic hyperarousal, perceptual disturbances, flashbacks, and even psychosis in those with particularly disturbing past experiences, e.g., childhood trauma or abuse (71, 72). A systematic review by Farias et al. (70), found that adverse events in meditation-based practices included psychosis, delusional events, fear and traumatic flashbacks and a population based survey of 434 people in the US suggested that approximately one third of people had experienced a meditation-related adverse effect such as re-experiencing of trauma, anxiety, emotional sensitivity and functional impairments (73). Surprisingly, approximately 80% of those experiencing adverse effects reported they were still “glad” to have meditated.

It is clear that a “one size fits all” approach to mindfulness meditation carries risks, particularly to people who already suffer from psychiatric illnesses or those who have had previous negative life-experiences (71). The British Psychological Society refers to guidance for monitoring and reporting of harm or side-effects in patients with psychosis (74), and this might also be applicable to people with persistent pain, with or without mental illness.

## Discussion

### Mindfulness to promote the “healing journey”

A salutogenic view of the benefits of mindfulness to promote living well with persistent pain could encompass an ecology of wholeness and the context of a person's “healing journey” (19, 75). Toye et al., (76) conducted a meta-ethnography that synthesized the findings of 195 qualitative studies exploring the experience of people living with persistent, non-malignant musculoskeletal pain that identified key elements of a health intervention to assist people on their “healing journey”:

1. Validating pain through meaningful and acceptable explanations.
2. Validating patients by listening to and valuing their stories.
3. Encouraging patients to connect with a meaningful sense of self, to be kind to themselves, and to explore new possibilities for the future.
4. Facilitating safe reconnection with the social world.

Toye et al. concluded that people in pain should be encouraged to move forward *alongside* their pain, rather than focusing on expectations of a cure; a model in which self-value, acceptance and recognition are central. Mindfulness is already being embedded in a variety of biopsychosocial interventions used to alleviate pain, thus promoting re-connection with a meaningful sense of self. This would enable people to explore new possibilities for the future.

### Mindfulness within a whole health delivery system

We advocate placing mindfulness at the core of interventions to aid recovery by improving a sense of coherence and empowering people to embrace their existence as a whole, giving their lives meaning and potential for fulfilment. In health care, mindfulness is seen as a psychological tool to aid self-management and is disconnected from many biomedical approaches (e.g., surgery and medication). Mindfulness is often at the core of community support activities e.g., yoga, that are rarely integrated with standard health care service delivery. Mindfulness has potential to develop health and well-being across core theoretical components underpinning interventions to promote the health of individuals, communities and nature (i.e., Whole Health perspective), such as those described by Kemp and Fisher (77); balanced mind, healthy body, connecting with people, connecting with nature, socio-structural factors, and sustaining behaviour change. Thus, we believe that encouraging mindfulness as an integrated lifestyle practice offers opportunities for people to use these techniques as adjuncts for many biopsychosocial interventions. Mindfulness skills could be developed through social prescribing of community-based support services for healthy living, and this could confer benefits in all aspects of life from activities of daily living to life enriching activities such as gardening, arts and crafts, walking, swimming, etc. Locating mindfulness as a lifestyle practice sits more comfortably within a whole-systems approach where people move forward in their journey towards better health through validation, acceptance, empowerment and, ultimately, fulfilment.

### Future research

Presently, there is tentative, low quality evidence that mindfulness is beneficial for pain (78). Recently, Moore et al., (79) have raised serious concern about methodological shortcomings of RCTs in the broader field of pain research resulting in low confidence in evidence for many analgesic treatments. The Medical Research Council's guidance suggests that attention be given to how interventions are used in the real world (i.e., their utility) including mediating factors, implementability, acceptability, feasibility of delivery and cost-effectiveness (80, 81). Thus, research evaluating baseline level of mindfulness skills, optimal “dose” and moderating influences of environmental factors may prove informative (31, 82). Attention also needs to be given to how best to capture holistic outcomes within the ecology of wholeness model without fragmenting this “wholeness” into a collection of discrete outcomes that are viewed as disconnected entities.

## Conclusion

Pain, especially when persistent, catalyses a loss of identity, a diminished sense of self, retreat from the world outside of the painful body and alienation and detachment from a meaningful life (83). Bullington (83) argues that rehabilitation must open up new possibilities of a life beyond or alongside pain through an enhanced sense of self.

By developing skills to attend, on a moment-to-moment basis, to happenings inside and outside of oneself mindfulness can rebuild a sense of cohesion and “wholeness”. In doing so, mindfulness can be used to instil a positive, proactive, approach to promote health through learning of a new sense of self and shifting focus away from the dominant biomedical narrative of deficit and opening new opportunities for a fulfilling life beyond, or alongside pain.

Our viewpoint is that mindfulness could be a core element of salutogenic approaches to promote health and well-being for people living with pain because mindfulness rebuilds the sense of cohesion fractured when pain threatens the future self. Mindfulness underpins an “ecology of wholeness” and could be a strategy used by people to mitigate the detrimental consequences of painogenicity. Greater credence should be given to the findings of research investigating the effects of mindfulness on people with pain in real-life settings that considers the influence of social, environmental, and economic factors using a broader set of outcomes including self-efficacy, sense of coherence and quality of life. This will shift the focus of evidence-gathering and expectations of rehabilitation from efficacy and pain to utility and outcomes associated with valuable and fulfilling lives.

## Data Availability

The original contributions presented in the study are included in the article/Supplementary Material, further inquiries can be directed to the corresponding author.
